# Hydrogel-Based Fluorescent Dual pH and Oxygen Sensors Loaded in 96-Well Plates for High-Throughput Cell Metabolism Studies

**DOI:** 10.3390/s18020564

**Published:** 2018-02-13

**Authors:** Shanshan Wu, Siying Wu, Zheyuan Yi, Fei Zeng, Weizhen Wu, Yuan Qiao, Xingzhong Zhao, Xing Cheng, Yanqing Tian

**Affiliations:** 1 Department of Materials Science and Engineering, Southern University of Science and Technology, No 1088 Xueyuan Road, Xili, Nanshan District, Shenzhen 518055, China; wus2008@aliyun.com (Sh.W.); wusiying0524@126.com (Si.W.); yizy@mail.sustc.edu.cn (Z.Y.); zengf@mail.sustc.edu.cn (F.Z.); wuwz@mail.sustc.edu.cn (W.W.); qiaoy@mail.sustc.edu.cn (Y.Q.); 2 Department of Physics, Wuhan University, Wuhan 430072, China; xzzhao@whu.edu.cn

**Keywords:** dual oxygen-pH sensing plates, oxygen respiration, extracellular acidification, high throughput analysis

## Abstract

In this study, we developed fluorescent dual pH and oxygen sensors loaded in multi-well plates for in-situ and high-throughput monitoring of oxygen respiration and extracellular acidification during microbial cell growth for understanding metabolism. Biocompatible PHEMA-co-PAM materials were used as the hydrogel matrix. A polymerizable oxygen probe (OS2) derived from PtTFPP and a polymerizable pH probe (S2) derived from fluorescein were chemically conjugated into the matrix to solve the problem of the probe leaching from the matrix. Gels were allowed to cure directly on the bottom of 96-well plates at room-temperature via redox polymerization. The influence of matrix’s composition on the sensing behaviors was investigated to optimize hydrogels with enough robustness for repeatable use with good sensitivity. Responses of the dual sensing hydrogels to dissolved oxygen (DO) and pH were studied. These dual oxygen-pH sensing plates were successfully used for microbial cell-based screening assays, which are based on the measurement of fluorescence intensity changes induced by cellular oxygen consumption and pH changes during microbial growth. This method may provide a real-time monitoring of cellular respiration, acidification, and a rapid kinetic assessment of multiple samples for cell viability as well as high-throughput drug screening. All of these assays can be carried out by a conventional plate reader.

## 1. Introduction

Dissolved oxygen (DO) measurement has attracted great attention because oxygen is a critical indicator for monitoring the cellular microenvironment, especially in understanding metabolism [1], tumor respiration studies [2], neuron cell research [3], and ecology applications [4]. Many kinds of diseases and cancers are associated with the mutual effect of superoxide dismutase (SOD) and reactive oxygen species (ROS) [5]. ROS are originated as a natural by-product of metabolism processes involving DO [6]. Further, cellular respiration is the process both prokaryotic and eukaryotic cells use to convert the energy contained in the chemical bonds of nutrients into ATP energy. Besides the significance of DO in vitro, monitoring and regulating the pH in culture medium are key steps in the successful operation of bioreactions as well. Hydrogen ion concentration (pH value)-responsive permeable factors on the cell surface are commonly investigated at cancer drug therapy [7]. Cellular pHs are closely related with cell metabolism, disease occurrence, the proliferation and apoptosis of embryonic cells and stem cells. It has been reported that the extracellular pH in certain cases becomes more acidic than the neutral pH of around 7.2 for normal tissues, to pH values or around 6.5 or even 5.5 [8]. On the other hand, the metabolic switch from oxidative phosphorylation to aerobic glycolysis in cancer cells—the Warburg effect—is one of the emerging hallmarks of cancer [9,10]. These two processes of oxidative phosphorylation and aerobic glycolysis are directly relevant to DO and pH levels.

There are several types of methods known for DO and pH value determination, including pressure, electrochemistry, and optical approaches. Optical assays are frequently used for rapid analysis in clinical setups and in laboratories for its convenient features. Fluorescence analysis is one of the major optical approaches, providing non-invasive, easily operated, disposable, and low cost assays [11] which plays a critical role in bioprocess monitoring and diagnosis. Some review articles have summarized current knowledge about pH sensors or oxygen sensors, including dual pH/oxygen sensors [12,13,14] Although many suitable fluorescent materials are available and some are even commercially available for DO and pH measurement [15,16,17,18,19,20,21,22,23,24,25,26,27,28,29], few of them were demonstrated to be capable or applicable for high throughput and in-situ monitoring of cell respiration and acidification using the widely applied multi-well plates used in many bio-labs [18]. Kocincova et al. reported the use of methacrylate-based pH-sensitive microbeads stained with 8-hydroxypyrene-1,3,6-trisulfonate (HPTS) as pH probes and oxygen-sensitive ruthenium-tris-4,7-diphenyl-1,10-phenanthrolinedi-(trimethylsilylpropanesulfonate) (Ru(dpp)_3_(TMS)_2_) microparticles in organosilica doped in polyurethane hydrogel as a dual pH and oxygen sensing material and deposited such dual sensors in 24 well plates for monitoring bacterial growth [18]. Further, Seahorse Bioscience has developed its XF96 metabolism instrument for oxygen respiration and extracellular acidification measurements [25]. The sensors in the XF96 instrument are a kind of polymeric gel-based sensor. For measuring cell respiration and acidification, Seahorse’s XF instrument uses a specific chip design to achieve its goal for high-throughput analysis of extracellular pH and oxygen measurements in their sophisticated and advanced instrument.

Our purpose is to prepare suitable hydrogels for dual pH and oxygen sensing to enable their direct use in 96 well plates acting as a type of representative functional multi-well plates as continuation of our previous work on the development of hydrogel-based sensors using thermal and/or redox polymerizations of polymerizable oxygen and pH probes [30,31,32,33]. Previously, we have developed some hydrogel sensors for individual pH [30] or oxygen [31,32], or dual pH and oxygen analysis [33]. Most of these were unfortunately unsuitable for direct use in the bottom of 96-well plates. Herein, we finely tuned the monomer mixture with a minimum amount of DMF and applied redox polymerization [34] for gelation of the monomer mixtures at room temperature on the bottom of 96 well plates for in-situ assays. Through careful control of the matrix’s composition, uniform sensing thin films were deposited on the bottoms of 96-well plates. The sensing performance was characterized in detail. Further the functionalized plates were used to monitor microbial cell respiration and extracellular acidification in *E. coli* with and without antibiotic drug treatments, demonstrating the capability of the application of the devices using an ordinary plate reader.

## 2. Materials and Methods 

### 2.1. Materials and Reagents

**Figure 1:**
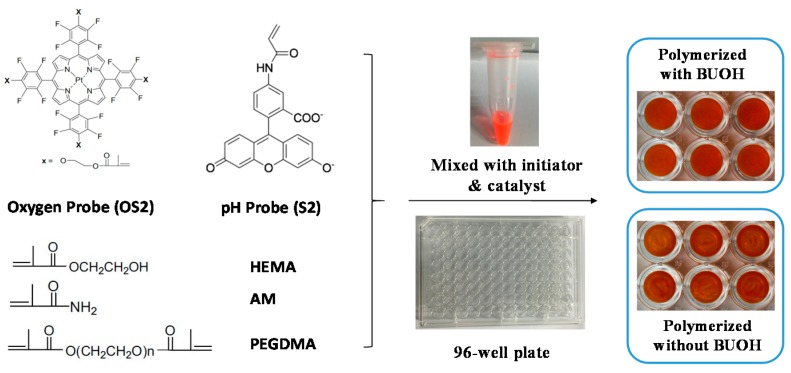
A simple schematic drawing for the preparation of 96-well sensing gel plate and the photos of the formed gels in a 96-well plate. BUOH indicates butanol or butyl alcohol.

All chemicals and solvents were of analytical grade and were used without further purification. Glucose, glucose oxidase, ethanol, DMF, dimethyl sulfoxide (DMSO), 2-hydroxyethyl methacrylate (HEMA), acrylamide (AM), poly(ethylene glycol) dimethacrylate (PEGDMA, Mn ≈ 550), and 1,1,4,7,10,10-hexamethyltriethylenetetramine (HMTETA) were commercially available from Sigma-Aldrich (St. Louis, MO, USA) and were used without further purification. The oxygen probe (OS2) and pH probe (S2) were prepared according to known procedures [31,33] and their structures are given in Figure 1. Doubly distilled water was used for the preparation of buffer solutions. A series of standard Britton-Robison pH buffers (BR, H_3_PO_4_-HOAc-H_3_BO_3_) ranging from pH 3.0–9.0 were prepared with 40 mM H_3_PO_4_, HOAc, and H_3_BO_3_ adjusted by NaOH (200 mM). Calibration gases (nitrogen and oxygen, each of 99.99% purity) were purchased from Shenzhen Huatepeng Special Gases Cooperation (Shenzhen, China). *E. coli K12* was purchased from ATCC (25404, ATCC, Manassas, VA, USA).

### 2.2. Instruments

A digital pH meter (Thermo Electron Corporation, Beverly, MA, USA) was used for pH measurements. A gas manipulator (Alicat Scientific Instrument, Tucson, AZ, USA, measuring error: ±1%) was used for controlling the exact gas percentage. All measurements of sensing behaviors were carried out at an atmospheric pressure (101 kPa) and room temperature (22 ± 1 °C). All the performances of multi-well plate sensing hydrogels were read by a plate reader (Cytation 3, BioTek, Winooski, VT, USA). A Lambda 25 UV/Vis spectrophotometer (PerkinElmer, Shelton, CT, USA) was used to read the OD values of *E. coli* at 600 nm for density determination.

### 2.3. Fabrication of Dual-Sensing Films in 96-Well Plates

In this work, the hydrogel matrices consist of 2-hydroxyethyl methacrylate (HEMA), acrylamide (AM), and poly(ethylene glycol) (PEGDMA) which acts as a cross-linker for gelation. OS2 and S2 were chosen as an oxygen probe and a pH probe, respectively. Platinum(II) porphyrin (OS2) is a representative emission-based oxygen probe with four methacrylate units for being easily immobilized into polymer matrices. OS2 is a derivative of the highly efficient oxygen probe of Pt(II)*meso*-tetra(pentafluorophenyl)porphine (PtTFPP) with high photo-stability and sufficient triplet-triplet energy transfer to oxygen molecules. Fluorescein derivative (S2) exhibits pH-dependent fluorescence changes due to its multiple pH-dependent ionic equilibria, where only fluorescein dianion and monoanion emit strong fluorescence under basic conditions and the intra-ester isomer formed under acidic conditions does not emit.

According to our previous experience, probe concentrations of 0.5 and 1.0 mg/g in matrixes did not obviously affect the sensing performance [33]. Herein, the pH probe S2 (0.9 mg) was dissolved in the 1 mL mixture of HEMA/AM/PEGDMA with proper ratios (S2 stock solution); the oxygen probe of OS2 was dissolved in DMF with the concentration of 3.6 mg/mL (OS2 stock solution); K_2_S_2_O_8_ in H_2_O (30 mg/mL) was prepared as an initiator for redox polymerization (initiator stock solution); 1,1,4,7,10,10-hexamethyltriethylenetetramine (HMTETA) was used as the catalyst in this system. Then pH probe S2 stock solution, oxygen sensor OS2 stock solution, initiator stock solution, and catalyst were mixed in suitable proportions to make the final stock solutions of the dual probes with concentration of S2 at 0.5 mg/g and OS2 at 1 mg/g in the matrices. In optimized conditions, the ratios of OS2 stock:S2 stock:initiator stock:HMTETA were 16:9:9:4 by volume. The well-mixed solution was dispensed by a micropipette onto the walls of a 96-well plate (40 μL for each well). To get a flat and uniform film, a layer of butanol (50 μL), which is almost immiscible with the gel precursors, was added on the top of the sensor solution with two functions: (1) the butanol layer acts as a sealing layer to avoid the interaction of the sensor precursors with air to speed up the radical polymerization and (2) the thin layer of butanol eliminates the tension effect of the sensor mixture in the well to get a smooth film. The mixed monomers were then allowed to cure for 1 h at room temperature. After that the butyl alcohol was removed by pipetting and the wells were washed with ethanol and then BR buffers (pH 3.0 and pH 7.0) before use.

### 2.4. Optimization of the Sensing Films

To enhance the sensing activities of the hydrogel films, we have explored the effects of the matrix’s components, mainly HEMA, AM, and PEGDMA, on the sensitivity through an optimization of their ratios. Hydrogel sensing films were prepared in 96-well plates as mentioned above in Section 2.3. Five films (F1–F5) with variable weight ratios of HEMA and AM (Table 1) were prepared with a fixed percentage (10%) of the PEGDMA crosslinker. Oxygen and pH titrations were used for optimization of the ratios of HEMA and AM. Another four films (F6–F9) were also prepared by changing the weight ratios of PEGDMA with the fixed optimized proportion of HEMA and AM for further optimizing sensing matrix.

### 2.5. Thickness Measurements of the Sensing Hydrogels

**Figure 2:**
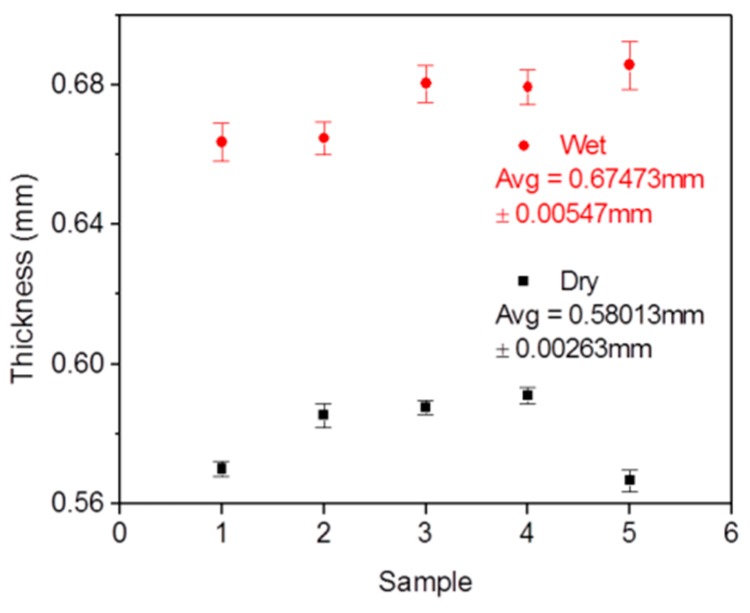
Thickness of five membranes in wet and dry conditions.

Thickness measurements for the gels were carried out under both wet and dry conditions by using a microcaliper. When measured wet, films were removed from the plate after polymerization, and their thickness was measured immediately. These fresh films were then put in a Petri dish at room-temperature for 3 days to dry slowly. Then the thicknesses were measured again. All the measurements were taken at the center of each film. Five samples were tested at each a condition. Average thicknesses and standard deviations are given in Figure 2.

### 2.6. Characterization of the Sensing Behaviors of the Hydrogels in 96-Well Plates

All the hydrogels were washed adequately with pH 7.0 BR buffer before the measurements. Fluorescence property of the hydrogels in multi-well plate was read by a plate reader. Emission spectra collected from 520 nm to 680 nm were measured at the excitation wavelengths of 405 nm (for oxygen probe) and of 488 nm (for pH probe), respectively.

### 2.7. Photostability Test and Ionic Influences

The photostability of O_2_ probes and pH probes were monitored at 645 nm with an excitation of 405 nm and at 525 nm under the excitation of 488 nm, respectively. As shown in Figure S1 in the Supplementary Materials, after four hours of continuously exposure, good stability with less than 10% decomposition was observed. The influence of ions on emission changes of the two probes was tested by adding a few kinds of biologically relevant cations at their physiological concentrations to the liquids. We did not observe any influence on the oxygen concentrations and pHs (Figure S2 in the Supplementary Materials).

### 2.8. pH Responses

pH values from 3.0 to 9.0 were chosen to characterize the pH responses of the sensing films. Before titration, each well was previously repeatedly washed by pH 3.0 and pH 9.0 buffer for several times. The titration was carried out from basic to acidic conditions. Each well was washed at least three times when changing buffers. Titrations were performed in duplicate to get the average value. The hydrogel was excited at 488 nm, and the emission spectra were measured from 510 to 660 nm.

### 2.9. Oxygen Responses

DO concentrations were adjusted by saturating the buffers or medium using the gas bubbling approach. When we collected the spectra of the hydrogels in the 96 well plates using the plate reader, we found the intensities at 645 nm changed fast because of the oxygen exchange with air, which may result in significant experimental calibration errors. Thus we did not collect the spectra of the oxygen probe, and instead, we just measured the intensities at 645 nm to avoid experimental errors.

### 2.10. E. coli Culture 

*E. coli K12* was cultured in liquid Lysogeny Broth (LB) medium at 37 °C with shaking at 200 rpm for 12 h. The cell density of *E. coli* in LB broth was estimated by measuring the optical density at 600 nm, which is linearly proportional to *E. coli* cell density when OD_600_ is in the range of 0.1 to 1.0. A value of 1 indicated a cell density of 5.0 × 10^8^ colony-forming unit per milliliter (CFU/mL), based on calibrations of the UV–vis spectrophotometer. Appropriate dilutions were made by using fresh LB to achieve the desired initial cell densities for oxygen respiration and cellular acidification experiments.

## 3. Results and Discussion

### 3.1. Sensor Preparation in Multi-Well Plates

A redox polymerization [34] method was used to prepare the multi-well hydrogel sensor plates, and no thermal treatment is needed for this polymerization. We also finely tuned the sensor monomer composition to minimize the use of DMF, which is a necessary solvent for OS2. By minimizing the use of DMF, the precursors for sensing film formation have no influence on the optical properties of the polystyrene of the 96-well plate bottoms. A schematic procedure for the hydrogel preparation in a 96-well plate was given in Figure 1. The concentrations for oxygen and pH probes for sensor hydrogel preparation were 1 mg/g and 0.5 mg/g, respectively, in the gel precursors. Premixed hydrogel precursor solutions (40 μL) were added to each micro-well and polymerized for 1 hour at room temperature. Two groups of cured gels in a 96-well plate were presented, which were polymerized with and without covering a layer of butyl alcohol, respectively. Gels appeared to have a lot of winkles in the micro-wells when polymerized without the butanol layer, while the films prepared with a layer of butyl alcohol were flat and uniform. The thickness of the flat films is about 0.675 ± 0.005 mm in wet condition and 0.58 ± 0.003 mm in dry condition (Figure 2).

**Figure 3:**
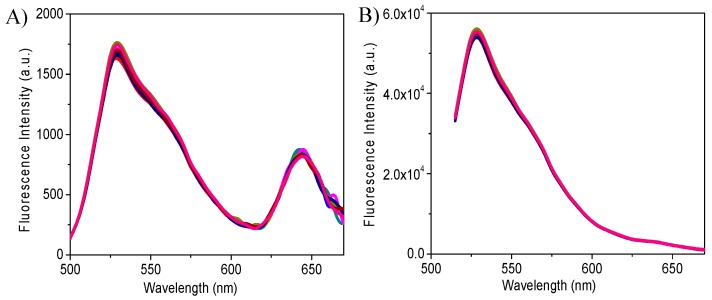
Fluorescence properties of nine fresh sensing gels in a 96-well plate in pH 7.0 BR buffer. (**A**) Excited at 405 nm and (**B**) Excited at 488 nm. The emission peak at 525 nm was attributed to the pH probe of S2; the emission peak at 645 nm was from the oxygen probe of OS2.

The fluorescence properties of a group of sensing gels (nine gels prepared in different wells) excited at 488 nm for pH sensor’s emissions only and excited at 405 nm for both the pH sensor and oxygen sensor are shown in Figure 3. Results showed that the fluorescence properties of the nine different films were almost the same, indicating the high reliability and repeatability of this approach and the potential high throughput applications of the sensor gels in 96 well plates.

### 3.2. Optimization of Sensing Hydrogels

To achieve sensitive sensing hydrogel films for biological studies, biocompatible copolymers of PHEMA-co-PAM were chosen as the matrices [35,36]. For achieving stable hydrogel, a dimethacrylate moieties-containing PEGDMA was used as a cross-linker to make the films robust. Since the matrix’s compositions may potentially affect the sensors’ behaviors, some optimization experiments were performed to get the best proportions of HEMA, AM and PEGDMA for achieving high sensitivity.

Firstly, five films (F1–F5, Table 1) with variable weight ratios of HEMA and AM were prepared with a fixed percentage (10%) of PEGDMA. Uniform films F1–F4 were obtained, while with the increase amount of AM to 35%, the film’ quality for F5 was poor. Most likely, the high composition of PAM easily resulted in crystallization of the film hindering the hydrogel formation. Additionally, the oxygen sensitivity represented by using I_0_/I_100_ among the three films F1, F2, and F3 for DO were all round 5.7~5.95 (Table 1), where I_0_ is the emission intensity at 645 nm under deoxygenated condition and I_100_ is the emission intensity under oxygenated condition. Among the films of F1–F4, F4 showed the lowest sensitivity to DO with I_0_/I_100_ at 1.54, which is also the worst one for pH sensing by comparing the value of I_pH9.0_/I_pH3.0_, showing that the film with higher AM composition is not better for higher oxygen permeability or ion permeability. Since the F3 film showed the highest pH sensitivity, for the later studies, the ratios of HEMA and AM of F3 were fixed at 85% and 15% by weight when optimizing PEGDMA (F6–F9). It was found that films could not be formed when the cross-linker PEGDMA was less than 5 wt. % of the sum of HEMA and AM. DO sensitivity increased with increasing PEGDMA from 5% to 10%, and decreased when PEGDMA was more than 10%. This observation indicated that most likely the oxygen permeability is limited by the gel density and also the compositions of PEG segments. However, the sensing ability to pH was continuously strengthened with the increase of the cross-linker PEGDMA from 5% to 20 wt. %, showing the ion permeability was not inhibited by the PEG segment or crosslinking degrees. Considering the sensing abilities of both DO and pH, the compositions of F3 with 85% HEMA, 15% AM, and 10% of PEGDMA by weight was chosen as the optimized one (Table 1). Thus, all the hydrogels were prepared according to the protocol of F3 in the follow-up work.

### 3.3. Typical Oxygen Sensing for DO

**Figure 4:**
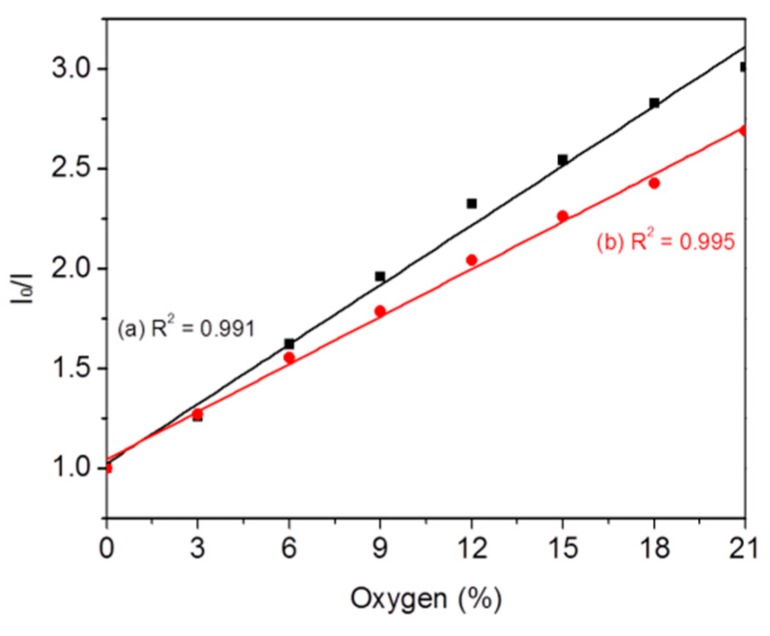
Response of the sensing gels to dissolved oxygen in a plate reader. Oxygen partial pressure was changed from 0 to 21% of atmosphere pressure. (**a**) fresh gel; (**b**) rehydrated gel (after one week). The intensity (I) was measured at 645 nm.

A mixture of oxygen and nitrogen gas was used to saturate aqueous solutions to tune DO concentrations by bubbling into a plate reader. Fluorescence intensities at 645 nm were measured at different DO concentrations. Figure 4a shows DO responses of F3 with 0% of oxygen (deoxygenated condition) to 21% of oxygen (8.6 ppm in water at 23 °C, air saturated condition). Intensity ratios (I_0_/I) of the F3 followed the Stern-Volmer equation with a correlation coefficient (R^2^) exceeding 0.997 as given in Equation (1):

(1) $$\frac{I_{0}}{I}=\;1\;+\;K\mathrm{sv}\left[{\mathrm{pO}}_{2}\right]\;$$

where *K*_SV_ is the Stern-Volmer quenching constant. *I*_0_ and *I* are the steady-state fluorescence intensity at 645 nm measured in deoxygenated and oxygen containing solutions, respectively. [pO_2_] is the oxygen partial pressure in the nitrogen and oxygen mixture gas.

Fluorescence intensities of the OS2 probe decreased with the increase in DO concentration. It is well-known that PHEMA-based hydrogels have excellent oxygen permeability for contact lenses [35,36]. The DO can interact efficiently with the sensor molecules in the gel as it swells significantly in water [33]. It was found that the fresh film has a better sensitivity than that of rehydrated film after one week. This is most likely due to the fact that the gel might keep on continuously crosslinking during the slowly drying process, since we only let the gels dry slowly in air at room temperature without using a strong drying condition. Therefore, we used the fresh hydrogels for further studies.

### 3.4. pH Sensing

**Figure 5:**
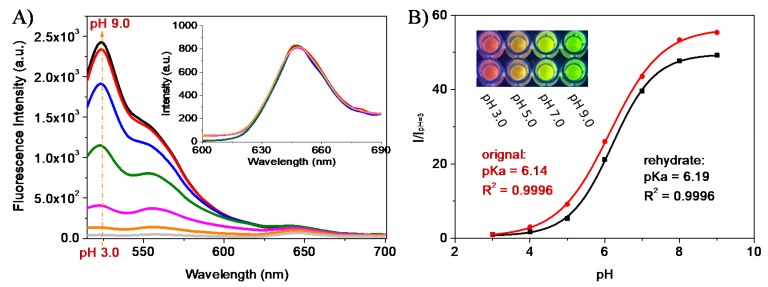
pH titration of the film F3 in a 96-well plate via a plate reader. (**A**) Fluorescence scan of F3 in a series of BR buffers excited at 488 nm; the insert figure in A is for the emissions from the oxygen probes excited at 540 nm. (**B**) Boltzmann curves of fresh and rehydrated gels (after one week). The insert in B shows the color changes at different pHs illuminated by a 365 nm LED light and taken by a color camera. Two parallel experiments were performed using fresh hydrogels.

pH responses were studied from pH 3 to 9 using the fresh and rehydrated films. Figure 5A showed the pH responses of a fresh film excited at 488 nm for S2 and 540 nm for OS2. The emission peaks of the two probes are well separated, showing that there were no cross-talks between pH and oxygen probes. This alleviates the interferences between the two probes. The sensing performance followed the Boltzmann responses (Equation (2) and Figure 5B). Green emission decreased with the decease of pHs, similar to those of other fluorescein-containing probes. As shown in the insert figure of Figure 5A, oxygen probe’ emission was not affected by the pH values. The p*K*_a_s for the sensing hydrogels were calculated to be 6.14 for fresh film and 6.19 for the rehydrated film after one week drying at room temperature. Similar to the oxygen responses, rehydrated films showed slightly decreased sensitivity (Figure 5B):

I = I_max_ + I_min_ × [10^pKa-pH^/(1 + 10^pKa-pH^)], (2)

where I is the fluorescent intensity at 525 nm, I_max_ is the maximum signal at pH 9.0, and I_min_ is the minimum signal at pH 3.0.

The insert in Figure 5B showed the emission color changes at a few typical pH values. The hydrogels contain pH probes with green emissions and oxygen probes with red emissions. At pH 9, strong green emission was observed; while at pH 3, because of the disappearance of green emission, red emission was observed.

### 3.5. Biocompability Test of the Sensor Hydrogels in the Wells

**Figure 6:**
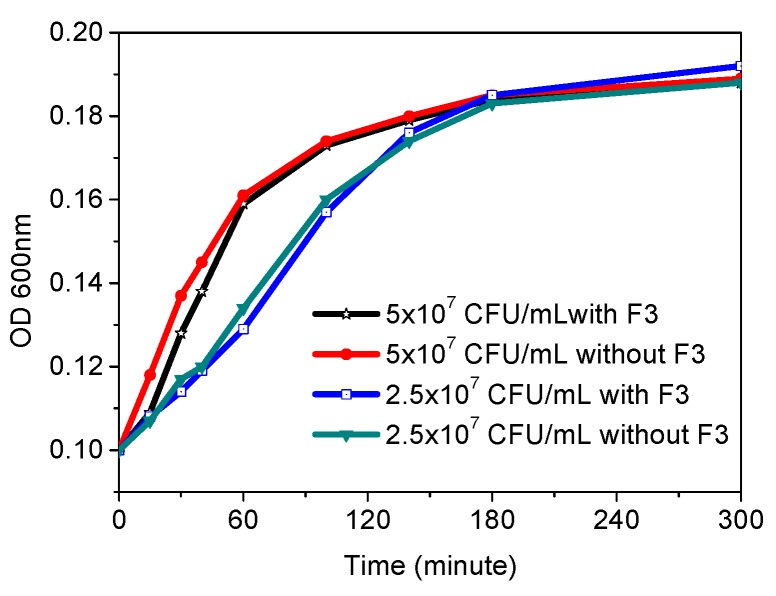
Time dependent OD_600_ of 2.5 × 10^7^ and 5 × 10^7^ CFU/mL of *E. coli* with and without F3 film at 37 °C.

For bioapplications, a toxicity study was required for proving the biocompatibility. Parallel experiments were conducted in the multi-well plate under the same conditions with and without the sensing hydrogels. One hundred μL of *E. coli K12* with densities of 2.5 × 10^7^ and 5 × 10^7^ CFU/mL in LB broth were seeded into each micro-well with and without sensing gels, and cultured in the plate at 37 °C for 5 h. The absorbance was periodically monitored at a wavelength of 600 nm (OD_600_) by a plate reader. Results showed that time dependent OD_600_ were not affected by the hydrogel-based sensors in the wells (Figure 6) for 5 h’ cell culture, showing the sensing hydrogels’ excellent biocompatibility.

### 3.6. In-Situ Monitoring of the pH and Oxygen Changes during the Growth of E. coli K12

**Figure 7:**
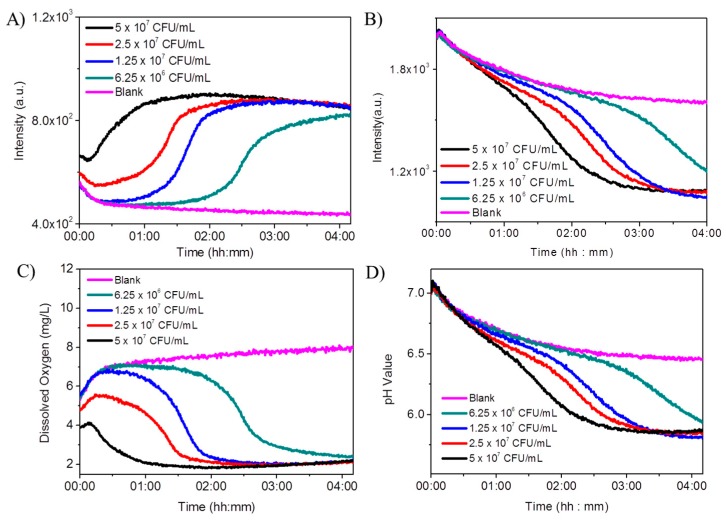
DO and pH monitoring of *E. coli K12* using a 96-well sensing gel plate. Fluorescence intensity changes were monitored at 645 nm with an excitation at 405 nm (oxygen probe, **A**), and at 525 nm under the excitation of 488 nm (pH probe, **B**) during the growth of *E. coli* with different initial inoculum at 0 (blank), 6.25 × 10^6^, 1.25 × 10^7^, 2.5 × 10^7^ and 5 × 10^7^ CFU/mL. (**C**,**D**): The calculated time dependent DOs and pHs from A and B, respectively by referring their corresponding calibration curves as showing in Figure 4 and Figure 5B. The blank is from a well only with sensors without *E. coli*.

*E. coli K12* was cultured in LB broth at 37 °C with shaking at 200 rpm for 12 h. Cells were gradually diluted with LB broth to 5.0 × 10^7^ CFU/mL, 2.5 × 10^7^ CFU/mL, 1.25 × 10^7^ CFU/mL and 6.25 × 10^6^ CFU/mL. Then 100 μL of each diluted sample in LB medium was seeded into the well with sensing gels, and the same volume of LB broth without cells was set as the blank control. In order to prevent the exchange of oxygen in the media with air, 100 μL of mineral oil was used to seal the well to avoid the exchange of oxygen in cell culture medium with air. The use of mineral oil for oxygen consumption is popularly applied [37]. Emission measurements at 645 nm and 525 nm were carried out to record the oxygen and pH changes during the cell growth with excitation wavelengths of 405 nm for oxygen probe and 488 nm for pH probe, respectively. It was observed that the oxygen probes’ emissions increased much faster at higher cell densities, indicating that the oxygen was consumed much faster at these higher densities (Figure 7A). After the DO in the medium was consumed completely, the fluorescence intensities did not change further. A slight decrease of the fluorescence intensities at 645 nm at the early stage of the experiments was observed, which was attributed to the temperature effect.

A very significant temperature effect was observed for the pH probe. Clear decreases of the fluorescence intensities at 525 nm were observed within the first 1 h of the experiments. After that a slight fluorescence decrease was observed for the blank sample, along with significant decreases of the fluorescence intensities with cells. Samples with higher cell densities produced faster intensity decreases, showing faster acidification rates. It was observed the pH changes were much slower than oxygen changes. This is due to that the fact LB medium has significant buffering capability to delay the pH changes. On the other hand, pH changes were observed after the DO was consumed completely, showing cells can still undergo metabolism. This is because that H^+^ ions were translocated outwards across the plasma membrane of *E. coli* by a respiratory pulse in the anaerobic conditions [38].

The fluorescence signals as shown in Figure 7A,B were transferred to DO concentrations and pHs by referring their corresponding calibration curves as shown in Figure 4 and Figure 5B, which were given in Figure 7C,D. Therefore, the hydrogel-based 96 wells with sensors showed the capability for simultaneously monitoring the pH and oxygen changes at cellular microenvironments. Since the gels were incorporated at the bottom of the 96 wells, high throughput analysis of cellular environment can be easily achieved. However, because of the significant temperature effect and also the possible photo-bleaching effect for the pH probes based on fluorescein, for accurate analysis of the pH and oxygen changes, new sensor formats including the probes, matrices, and/or thicknesses of films with less temperature effects and better photostability needed to be considered and chosen.

### 3.7. Sensing Films for Antibiotic Test

**Figure 8:**
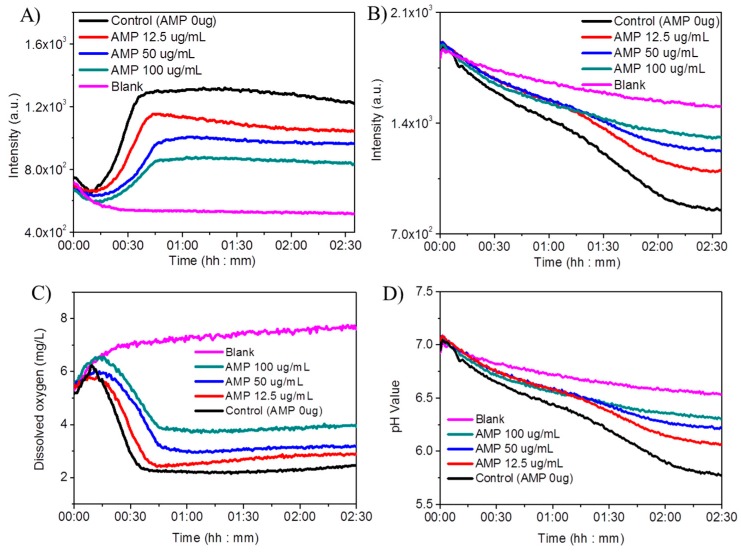
Antibiotic (ampicillin, AMP) test against *E. coli K12* with sensing films in a 96-well plate. Fluorescence intensity changes monitored at 645 nm with an excitation at 405 nm (oxygen probe, **A**) and at 525 nm under the excitation of 488 nm (pH probe, **B**). DO concentrations and pH changes corresponding to A and B, respectively, which was calculated by referring their individual calibration curves as plotted in Figure 4 and Figure 5B. The blank is from a well only with sensors without *E. coli*. Control is a well with *E. coli* however without AMP.

The influence of antibiotics on the cell metabolic activity, herein oxygen consumption and pH, was investigated by using ampicillin as an example. Ampicillin (AMP) is a β-lactam antibiotic which could act as a competitive inhibitor of the enzyme transpeptidase for cell-wall-forming, and ultimately leads to cell lysis [31]. For this study, *E. coli K12* cells were cultured with an initial OD_600_ of 0.1 under various concentrations of ampicillin (12.5, 25 and 50 μg/mL) under aerobic condition at 37 °C with shaking in the 96-well sensing plate. AMP concentration dependent inhibition of oxygen consumption was observed (Figure 8A,C). Higher AMP concentration resulted in more significant oxygen consumption inhibition. A similar effect was also observed for the pH changes (Figure 8B,D). Higher AMP concentration resulted in more significant cellular acidification inhibition. Hence, our studies indicated our sensing materials have potentials for understanding of the antibiotics influence on cell metabolic activities and the evaluation of the antibiotics activities.

## 4. Conclusions

New hydrophilic PHEMA-co-PAM hydrogel-based oxygen/pH dual sensing films were fabricated on the bottom of commercial multi-well plates through redox polymerization. The sensors’ sensitivity was optimized by adjusting the proportions of the hydrogel matrix components PHEMA, PAM, and the cross-linker PEGDMA. The final composition for good sensing performance was found to be a weight ratio of HEMA:AM:PEGDMA of 85:15:10. To get flat and uniform hydrogel films in the 96 well pates, a thin layer of butyl alcohol was placed on the top of the mixed monomeric precursors for gel formation in each micro-well. The sensor films with the aid of butyl alcohol are quite flat and smooth. The hydrogels could be reused and were robust enough to be washed, dried, rehydrated and stored in air for at least 1 week. Such functional 96-well plates were used for simultaneously monitoring the changes of pH and oxygen during the growth of *E. coli*. Clear cell density dependent oxygen consumption and extracellular acidification rates were observed by using the sensor gels. Further, the sensors provided the capability for studying the effects of antibiotics on cell oxygen respiration and cellular acidification. Although the current sensor formats still exhibited photo-instability and temperature effects during the measurements, our results demonstrated herein a new avenue to broaden the oxygen and pH sensor’s applications in biological fields for in-situ and high throughput analysis of pH and dissolved oxygen in cellular micro-environments. It is expected that this type dual-functional biocompatible sensing hydrogel can be used for understanding of cell growth, proliferation, and cell metabolism.

## Figures and Tables

**Table 1:** Compositions of sensing hydrogel films of F1–F9 and their sensitivity.

Films	HEMA ^a^	AM ^a^	PEGDMA in the Total Weight of HEMA and AM	Film-Forming	Sensitivity to DO ^b^ (I_0_/I_100_)	Sensitivity to Ph ^c^ (I_pH9.0_/I_pH3.0_)
**F1**	100	0	10	yes	5.73	17.3
**F2**	95	5	10	yes	5.95	18.0
**F3**	85	15	10	yes	5.83	21.4
**F4**	75	25	10	yes	1.54	11.3
**F5**	65	35	10	no	-	-
**F6**	85	15	2.5	no	-	-
**F7**	85	15	5.0	yes	4.28	17.6
**F8**	85	15	15	yes	3.39	22.3
**F9**	85	15	20	yes	2.31	24.5

^a^ Weight ratios of HEMA/AM in the gel films. ^b^ Fluorescence intensity ratios between those with 100% nitrogen and with 100% of oxygen. I_0_ is the emission intensity at 645 nm under deoxygenated condition; I_100_ is the emission intensity under oxygenated condition. ^c^ Fluorescence intensity ratios between those at pH 9.0 BR buffer and at pH 3.0 BR buffer. I_pH9_ is the emission intensity of 525 nm at pH 9; I_pH3_ is the emission intensity of 525 nm at pH 3.

## Supplementary Materials

The following are available online at http://www.mdpi.com/1424-8220/18/2/564/s1, Figure S1: The photostability of O_2_ sensor and pH sensor. Emission intensity of oxygen probes was monitored at 645 nm with an excitation of 405 nm; emission intensity of pH probes was monitored at 525 nm with an excitation of 488 nm., Figure S2: dissolved oxygen changes (A), and pH changes (B) for the sensing films in the presence of various biological cations at their physiological concentrations in B.R. buffer (pH 7.4): KCl (5.0 mM), NaCl (15 mM), MgSO_4_ (2.5 mM), CaCl_2_ (0.5 mM), FeCl_2_ (18 mM), CuCl_2_ (16 mM) and MnCl_2_ (0.9 mM).
