# Supplementary material for: Hydrogel-Based Fluorescent Dual pH and Oxygen Sensors Loaded in 96-Well Plates for High-Throughput Cell Metabolism Studies
Source: Sensors (Basel). 2018 Feb 13;18(2):564. doi: 10.3390/s18020564 (PMC5855129; doi:10.3390/s18020564)
Supplement: Supplementary file 1 [file sensors-18-00564-s001.pdf]

Supporting Information

Hydrogel-based Fluorescent Dual pH and Oxygen Sensors Loaded in 96-well Plates for High-throughput Cell Metabolism Studies

Shanshan Wu [1,2,†], Siying Wu [1,†], Zheyuan Yi [1], Fei Zeng [1], Weizhen Wu [1], Yuan Qiao [1], Xingzhong Zhao [2], Xing Cheng [1,*] and Yanqing Tian [1,*]

[1] Department of Materials Science and Engineering, Southern University of Science and Technology, No 1088 Xueyuan Road, Xili, Nanshan District, Shenzhen, Guangdong, 518055, China; cheng.x@sustc.edu.cn; tianyq@sustc.edu.cn
[2] Department of Physics, Wuhan University, Wuhan, Hubei Province 430072, China
* Correspondence: tianyq@sustc.edu.cn; Tel.: +0755-88018997
† equal contributors.

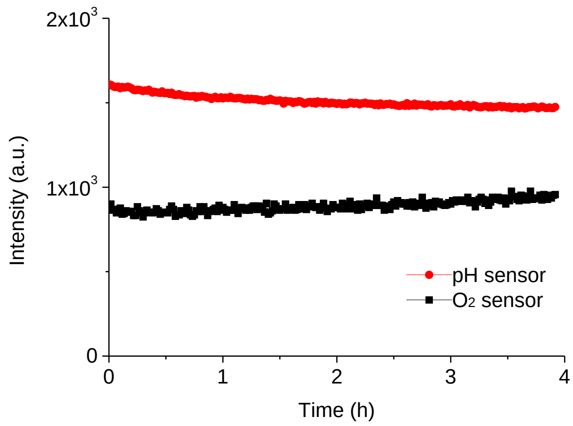

Figure S1. The photostability of O_2 sensor and pH sensor. Emission intensity of oxygen probes was monitored at 645 nm with an excitation of 405 nm; emission intensity of pH probes was monitored at 525 nm with an excitation of 488 nm.

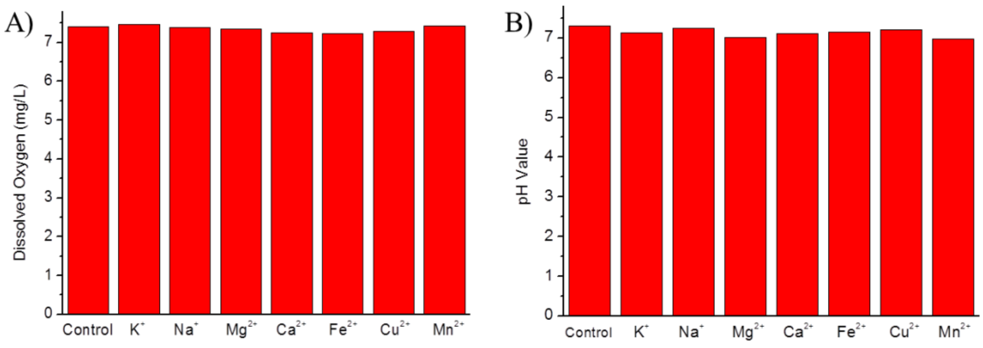

Figure S2 dissolved oxygen changes (A), and pH changes (B) for the sensing films in the presence of various biological cations at their physiological concentrations in B.R. buffer (pH 7.4): KCl (5.0 mM), NaCl (15 mM), $MgSO_4$ (2.5 mM), $CaCl_2$ (0.5 mM), $FeCl_2$ (18 mM), $CuCl_2$ (16 mM) and $MnCl_2$ (0.9 mM).
